# The Functional Examination of the Eye

**Published:** 1896-05

**Authors:** 


### Book Reviews.

The Functional Examination of the Eye. By John Herbert Claiborne, Jr., M. D., Adjunct Professor of Ophthalmology, New York Polyclinic ; Instructor of Ophthalmology in the College of Physicians and Surgeons, New York ; Assistant Surgeon to the New Amsterdam Eye and Ear Hospital, New York ; formerly Attending Surgeon, Northwestern Dispensary, Eye, Ear and Throat Department ; formerly Clinical Assistant to the Manhattan Eye and Ear Hospital ; author of Theory and Practice of the Ophthalmoscope. Octavo, pp. 96. With twenty-one illustrations. Price, $1.00. Philadelphia : The Edwards & Docker Co. 1895.

This little volume purports to make the subject of functional examinations of the eyes " clear beyond peradventure for those who shall read these pages. It is the author's purpose, in short, to present it in such a way that a student may follow the lines laid down and perform the examination with scientific and mechanical accuracy without further instruction." The author believes that "a fair success in teaching the subject for a number of years" has won for him "the right of presenting it," and that "the ventilation of the subject," "while it may be tedious," is "essential for those who know little of the subject or for those who have forgotten what they once knew;" and he has, therefore, intentionally adopted "a somewhat colloquial style" and some "graphic formulæ," and has consoled himself "that he has put the known facts of a dry subject in a pleasing form."

It will be seen by these quotations what the author proposes to do, and how well and in what improved style he thinks he has done it. If the student, however, expects to learn from this book how to do good work in the correction of errors of refraction, we fear he will be exceedingly disappointed. No man can succeed in getting the best results by the methods here taught.

